# Thick Darkness

**Published:** 1889-12-14

**Authors:** 


### Words of Consolation.

### THICK DARKNESS.

*(For Reading to the Sick.)*

Thick darkness! What alarming ideas do these words bring to the mind. A darkness that can be felt, such as was sent upon the Egyptians for their cruelty to the Hebrews; gross darkness, as it is called in Holy Scripture. A few years ago similar horrors were suffered by the unfortunate inhabitants of a small island in the Indian Ocean. A dead volcano, as it was supposed to be, suddenly came to life again and burst forth with fire and smoke, casting death and destruction on all around. Buildings, vegetation, and all living things were entirely destroyed, and such immense quantities of dust and earth were thrown up that the air was full of it for many miles round, and neither sun, nor moon, nor stars were seen for days. As described by the captain of an English vessel lying anchored near, the effects were terrific, the noise of the falling earth on the deck and the roaring of the elements were frightful, and were made tenfold worse by the thick darkness which surrounded them. The sailors dared not move lest they should fall overboard or be dashed to pieces by the raging elements. They suffered from hunger and thirst and the misery of despair. How their hearts leaped for joy when the first glimmer of light appeared! They felt new hope rise in their breasts, and as the tropical sun gradually pierced and dispersed the blackness around, they returned thanks with glad hearts to the Almighty for their preservation from the jaws of death. To take another instance nearer home, think of the confusion caused by a dense London fog. People can scarcely breathe for the thickness of the air; cold and miserable, they are bewildered by the darkness and wander out of their way, falling into untold dangers. But there is a worse darkness than either of those just described—the darkness of souls lost in sin and wickedness. Think, O, sinner, of the inky blackness by which you are surrounded. Like the lovely island in the summer sea, you are living in security, when suddenly the storm bursts—your evil nature which has been only slumbering during prosperity, bursts forth and overwhelms you. You grope after light, but it is the lurid glare which Satan holds out for your destruction. Oh, turn from it before it is too late and seek for the Sun of Righteousness, that sun which rises with healing in His wings. He is there shining above you, though you cannot see Him for the raging of the storm of passion in your heart, and the thick earthly cares and strife around you.

Or are you cold and dead in the raw fog of indifference and carelessness? or, maybe you are struggling against these hindrances and are striving to reach that blessed Sun which you know shines alike upon the evil and the good. Call upon your God with all your might. He will hear you, and cause the darkness around your soul to disperse. Not all at once, perhaps, but gradually the light of His countenance will pierce the gloom and scatter the selfishness and greed which wraps you round as with a cloak. Ah, poor bewildered soul, do you fear to step, lest you fall into unknown dangers. Jesus is at hand; trust in Him; He will lead you to the haven where you would be. He will calm your fearful heart, and give it peace; He will warm and comfort your cold, half-dead soul, and restore it to light and hope. He, your Saviour, bore the awful darkness which fell on the world as He hung for your sins on the dreadful cross. He can feel for your woes, and intercede with His Father and your Father for their relief.

O sinner, lift the eye of faith,
  To true repentance turning,
Bethink thee of the curse of sin,
  Its awful guilt discerning;
Upon the Crucified One look,
And thou shalt read as in a book,
  What will is worth thy learning.

